# Expression of mRNA Encoding Mcu and Other Mitochondrial Calcium Regulatory Genes Depends on Cell Type, Neuronal Subtype, and Ca^2+^ Signaling

**DOI:** 10.1371/journal.pone.0148164

**Published:** 2016-02-01

**Authors:** Nóra M. Márkus, Philip Hasel, Jing Qiu, Karen F. S. Bell, Samuel Heron, Peter C. Kind, Owen Dando, T. Ian Simpson, Giles E. Hardingham

**Affiliations:** 1 Centre for Integrative Physiology, University of Edinburgh, Edinburgh, EH8 9XD, United Kingdom; 2 School of Informatics, University of Edinburgh, Edinburgh, EH8 9AB, United Kingdom; 3 Centre for Brain Development and Repair, Institute for Stem Cell Biology and Regenerative Medicine, National Centre for Biological Sciences, Bangalore, 560065, India; IIBB/CSIC/IDIBAPS, SPAIN

## Abstract

Uptake of Ca^2+^ into the mitochondrial matrix controls cellular metabolism and survival-death pathways. Several genes are implicated in controlling mitochondrial Ca^2+^ uptake (mitochondrial calcium regulatory genes, MCRGs), however, less is known about the factors which influence their expression level. Here we have compared MCRG mRNA expression, in neural cells of differing type (cortical neurons vs. astrocytes), differing neuronal subtype (CA3 vs. CA1 hippocampus) and in response to Ca^2+^ influx, using a combination of qPCR and RNA-seq analysis. Of note, we find that the *Mcu*-regulating *Micu* gene family profile differs substantially between neurons and astrocytes, while expression of Mcu itself is markedly different between CA3 and CA1 regions in the adult hippocampus. Moreover, dynamic control of MCRG mRNA expression in response to membrane depolarization-induced Ca^2+^ influx is also apparent, resulting in repression of *Letm1*, as well as *Mcu*. Thus, the mRNA expression profile of MCRGs is not fixed, which may cause differences in the coupling between cytoplasmic and mitochondrial Ca^2+^, as well as diversity of mitochondrial Ca^2+^ uptake mechanisms.

## Introduction

Energized mitochondria are capable of taking up Ca^2+^ across the inner mitochondrial membrane into their matrix [1, 2]. This Ca^2+^ uptake has the capacity to modulate and buffer cytoplasmic Ca^2+^ signals, and is thought to also regulate several metabolic pathways within the mitochondria itself [3, 4]. Moreover, excessive mitochondrial Ca^2+^ uptake can contribute to cell death under certain pathological conditions [5–8].

In recent years, many genes involved in Ca^2+^ influx and efflux across the inner mitochondrial membrane have been discovered and added to more established candidates [1, 2, 9]. The gene encoding the pore-forming component of the potential-driven mitochondrial calcium uniporter (gene name: MCU) was recently discovered, and is an important mediator of mitochondrial Ca^2+^ uptake in many situations [10, 11].

MCU can form a complex with a close relative, MCUB (CCDC109B) which has a dominant negative effect on the complex with regard to Ca^2+^ permeability [12]. An additional protein, EMRE (*Smdt1*), has been reported to be required for Ca^2+^ channel activity mediated by Mcu [13]. The MCU complex is additionally regulated by a growing group of accessory proteins that play a key role in determining the exact dose response of the Mcu complex to extra-mitochondrial Ca^2+^. MICU1 and MICU2 are regulatory proteins that play distinct roles in ensuring that the MCU is largely inactive at low resting Ca^2+^ levels but becomes strongly permeant to Ca^2+^ at higher concentrations [14–18]. MICU3 represents a 3rd member of the MICU family, based on sequence homology, although its function remains unclear [16].

MCUR1 has also been identified as an important Mcu-interacting regulator of mitochondrial Ca^2+^ uptake [19–21]. *SLC25A23* is another protein implicated in controlling Mcu activity, potentially due to its interaction with MCU and MICU1 [22]. It is a Mg-ATP/Pi carrier that contains Ca^2+^ binding EF-hand domains and may act by sequestering MICU1 away from MCU. There is also considerable evidence that gene products other than Mcu can contribute to mitochondrial Ca^2+^ uptake. Principal among them is Letm1, identified in a siRNA screen as a mitochondrial Ca^2+^/H^+^ exchanger [23]. UCP2/3 has also been shown to influence mitochondrial Ca^2+^ uptake, and the dependence on UCP2, LETM1 and MCU may vary according to stimulus type [24, 25]. More recently, both TRPC3 and RYR2 have been reported to offer further Mcu-independent routes to mitochondrial Ca^2+^ uptake [26, 27]. Of course, Ca^2+^ efflux from mitochondria is also essential for homeostasis of matrix Ca^2+^ levels and in this respect, the Na+/Ca^2+^ exchanger NCLX (SLC8B1) is a likely mediator [28]. We refer to this non-exhaustive gene set as mitochondrial calcium regulatory genes (MCRGs).

Although the role of these gene products in controlling matrix Ca^2+^ levels is subject to considerable investigation, relatively little is known about what determines the overall expression profile of MCRGs. This is important to know, since variation in expression profile could impact on what route(s) of uptake are the most important, and on the nature of coupling between cytoplasmic Ca^2+^ and mitochondrial uptake. For example, we recently reported that synaptic activity causes the transcriptional repression of *Mcu*, and reduces coupling between cytoplasmic Ca^2+^ influx and mitochondrial Ca^2+^ uptake [8]. However, whether transcription of other MCRGs is influenced by Ca^2+^ signals is unclear. It is also not well understood how the basal expression profile of MCRGs varies with cell type. We have investigated these issues in the current study, focussing on neural cells (neurons and astrocytes), since mitochondrial Ca^2+^ overload is implicated in excitotoxic neuronal death, and physiological uptake into mitochondria thought to play a role in neuronal adaptive energy production in response to electrical activity [4]. Within this study we have focussed on MCRG expression at the mRNA level, and compared neural cells of differing types (cortical astrocytes vs. cortical neurons), neuronal subtypes (CA1 vs. CA3 hippocampus) and also studied the influence of cytoplasmic Ca^2+^ influx induced by electrical activity. The results revealed considerable heterogeneity in MCRG mRNA expression dependent on cell type, neuronal subtype and activity history.

## Results

### Cortical Astrocytes and Neurons Differ in Their MCRG mRNA Expression Profile

**Fig 1:**
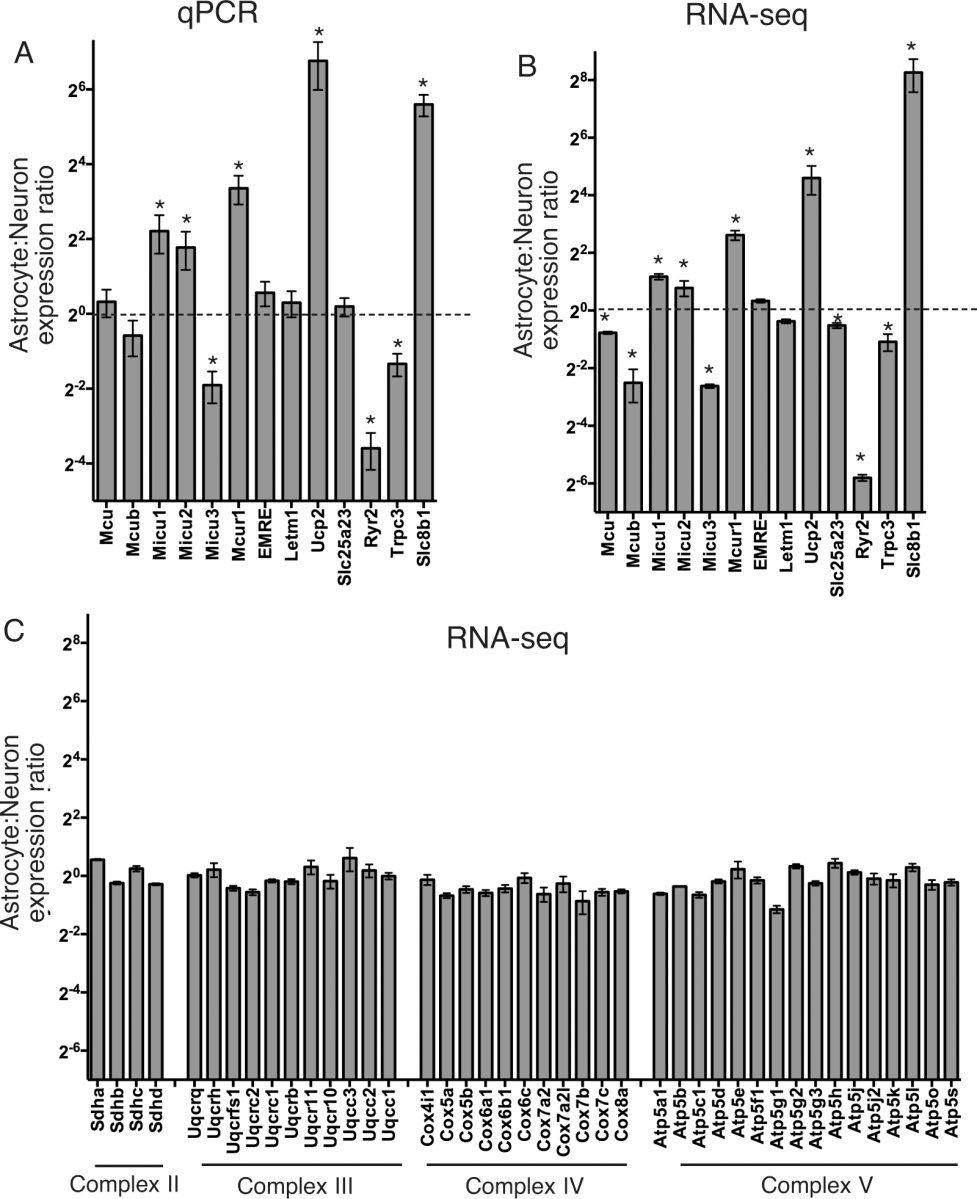
Mitochondrial calcium regulatory genes (MCRG) mRNA expression differs between cultured cortical neurons and astrocytes. **A)** qRT-PCR analysis of the indicated genes was performed in cortical neurons and astrocytes from paired cortical samples, plus mixed astrocyte/neuron culture for reference. Expression was normalised to 18s rRNA, the neuronal and astrocytic expression calculated relative to the mixed culture, and subsequently the astrocyte:neuron ratio calculated. * Exact p values (left to right) are 0.019, 0.030, 0.004, 0.002, 0.034, 1E-07, 0.007, 0.0002; alpha = 0.05; Student's one-sample t-test (n = 7 except Trpc3 and Slc8b1 (n = 6)). Note that the y-axis is on a log2 scale. The dotted line indicates a ratio of 2^0^ i.e. 1. **B)** RNA-seq analysis of neurons and astrocytes (n = 3) was performed and normalised read density of the indicated genes calculated, and the astrocyte:neuron ratio calculated. * Exact p values (left to right) are 0.001, 0.014, 2E-4, 0.023, 4E-4, 0.002, 0.043, 0.004, 0.005, 0.005, 5E-4; alpha = 0.05; Student's one-sample t-test (n = 3). **C)** Astrocyte: neuron ratios of RNA-seq reads for a selection of nuclear encoded mitochondrial proteins (components of ETC complexes II-V, n = 3).

We initially studied expression of MCRG mRNA by qPCR in highly enriched cortical neuronal cultures [29] (>98% NeuN+ neurons and <0.2% GFAP+ astrocytes) and highly enriched cortical astrocyte cultures [30] (>96% GFAP+ astrocytes). No significant differences in either *Mcu* or *Mcub* expression were found between neurons and astrocytes, normalising to 18s rRNA (Fig 1). However, differences in certain Mcu-regulatory genes were observed: neurons exhibited much lower expression of *Micu1*, *Micu2* and *Mcur1*, but considerably higher levels of *Micu3*. Of the other genes implicated in mediating mitochondrial Ca^2+^ uptake, Letm1 expression was similar in neurons and astrocytes, but *Ucp2* expression around 90-fold higher in astrocytes (Fig 1). Expression of other putative mitochondrial Ca^2+^ uptake genes, *Ryr2* and *Trpc3*, were also strongly different (Fig 1), however, since they are also expressed in other parts of the cell, the significance of these differences is less clear. Another difference is in the expression of *Slc8b1*, considerably higher in astrocytes than neurons.

To investigate MCRG mRNA expression in neurons vs. astrocytes using an alternative technique we performed RNA-seq on samples taken from neuronal and astrocytic cultures (biologically distinct from the samples analysed in Fig 1A). RNA-seq reads mapped to each of the MCRGs were normalised according to total read count, and then the relative read density in astrocytes compared to neurons was calculated (Fig 1B). We observed a qualitatively similar relative gene expression pattern using RNA-seq as with the qPCR technique (Fig 1A), although the RNA-seq analysis pointed to slightly lower Mcu levels in astrocytes than neurons. We also exploited the RNA-seq data set to compare the astrocyte:neuron ratios in MCRG mRNA expression to expression ratios of another set of nuclear-encoded mitochondrial genes: components of mitochondrial electron transport chain (ETC) complexes II-V (Fig 1C). As can be seen, overall there is little variation in expression levels of these genes at the mRNA level between astrocytes and neurons, indeed, the average ratio of all ETC genes is 0.91 ± 0.04 (n = 43), with a coefficient of variation (CV) of 0.27. There is a much larger heterogeneity in MCRG mRNA expression ratios (CV = 3.18), suggestive of a degree of cell-type specific diversity in mitochondrial Ca^2+^ handling.

### Hippocampal Subregions Express Different Levels of MCU

**Fig 2:**
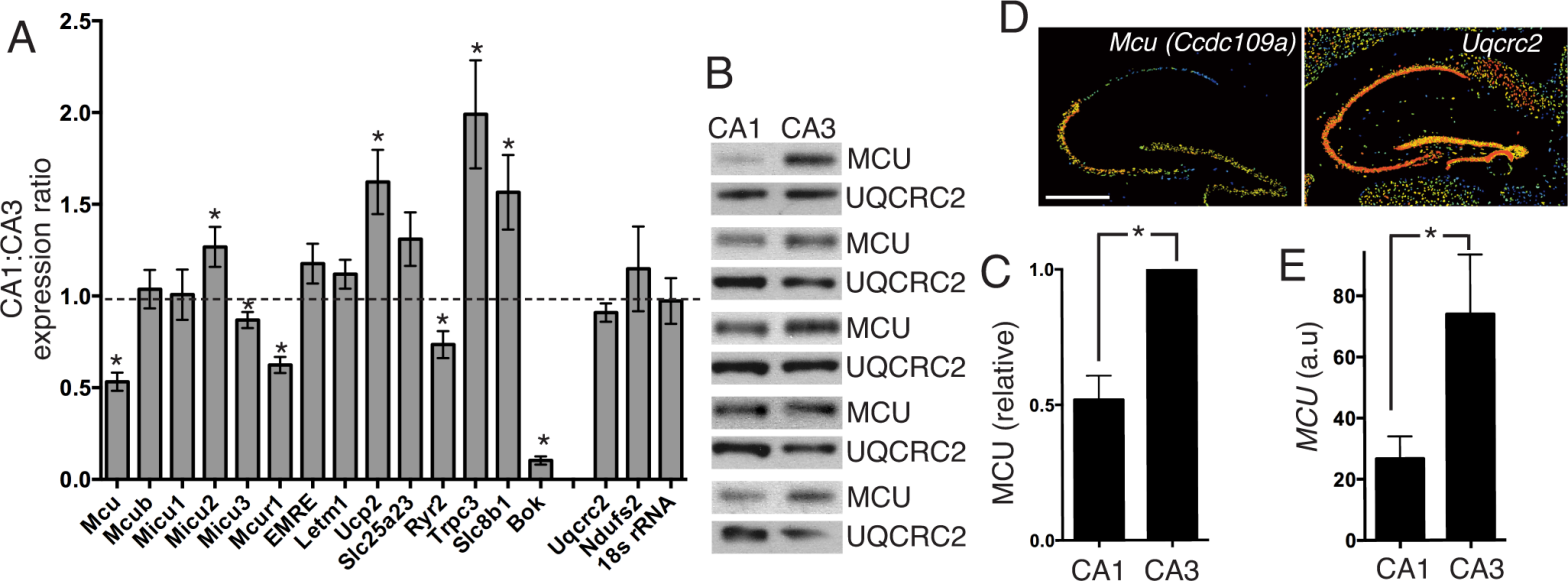
MCRG expression differs between CA1 and CA3 sub-regions of the hippocampus. Adult hippocampal slices were cut, and regions approximating to CA1 and CA3 were microdissected and analysed as paired material. **A)** mRNA expression was analysed and normalised to *Gapdh* and the CA1:CA3 ratio calculated of MCRGs. An additional gene, Bok, was analysed as a positive control as this is known to be enriched in CA3 over CA1. Additionally, control genes 18srRNA and nuclear-encoded mitochondrial ETC genes Uqcrc2 and Ndufs2 were analysed. * Exact p values (left to right) are 3E-06, 0.033, 0.024, 0.0001, 0.005, 0.005, 0.015, 0.021, 2E-08; alpha = 0.05; Student's unpaired t-test (n = 11 except Mcub (n = 9), Micu3, Mcur1, Trpc3, Bok, Uqcrc2, Ndufs2, 18srNA (n = 7), Slc8b1 (n = 10)). The dotted line indicates a ratio of 1. **B, C)** Western blot analysis of MCU protein expression in CA1 and CA3 regions from the same hippocampal slice, normalised to mitochondrial complex III protein UQCRC2 (C) shows analysis of the blots shown in (B). *p = 0.006, Student's t-test (n = 5 different animals). **D)** Example pseudocoloured images from the Allen Mouse Brain Atlas [32] at mouse.brain-map.org: experiments 69608519 (*Mcu*) and 72723 (*Uqcrc2*) are shown. Scale bar = 500 μm. Warm colours indicate higher expression. **E)** Graphical representation of microarray data relating to probeset A_23_P346405 (Mcu) from six individuals available at the Allen Human Brain Atlas at human.brain-map.org [33], showing only data on hippocampal regions CA1 and CA3. *p = 0.001; alpha = 0.05 (n = 6).

We next considered whether neurons from different brain sub-regions could have distinct MCRG mRNA expression profiles. We chose to compare MCRG mRNA expression profiles in the CA3 and CA1 subregions of the adult mouse hippocampus. One reason we chose these regions is the ease with which they can be anatomically distinguished, and another is the fact that differential gene expression in these regions has been reported [31]. Hippocampal slices were prepared from adult mice and the CA3 and CA1 regions from the same slices microdissected prior to RNA extraction, generating paired samples for comparison. To validate the approach, we measured expression of Bcl-2 related ovarian killer (*Bok*) whose expression is known to be enriched in CA3 compared to CA1 [31], and found this to indeed be the case (Fig 2A). We confirmed that, when normalizing to *Gapdh*, *18s rRNA* and ETC components *Uqcrc2* and *Ndufs2* were expressed at similar levels in CA3 vs. CA1 (Fig 2A). While a number of statistically significant, but nevertheless modest differences in MCRG expression were found, one striking observation was the difference in *Mcu* expression, which was found to be around 2-fold higher in CA3 than CA1 (Fig 2A). This difference was also observed at the protein level, as analysed by western blot, normalizing to ETC component UQCRC2 (Fig 2B and 2C). Interestingly, the differential expression of *Mcu* mRNA in CA3 vs. CA1 is also apparent in the Allen Mouse Brain Atlas [32] (Fig 2D, experiment ID: 69608519, *Uqcrc2* shown for comparison: experiment ID:72723, see mouse.brain-map.org) as well as in the Allen Human Brain Atlas [33] (Fig 2E, probe ID: A_23_P346405 (MCU)). Collectively these data raise the possibility that mitochondrial uniporter activity is substantially higher in CA3 neurons than those in CA1.

### Several MCRGs Are Regulated at the mRNA Level by Ca^2+^ Influx

**Fig 3:**
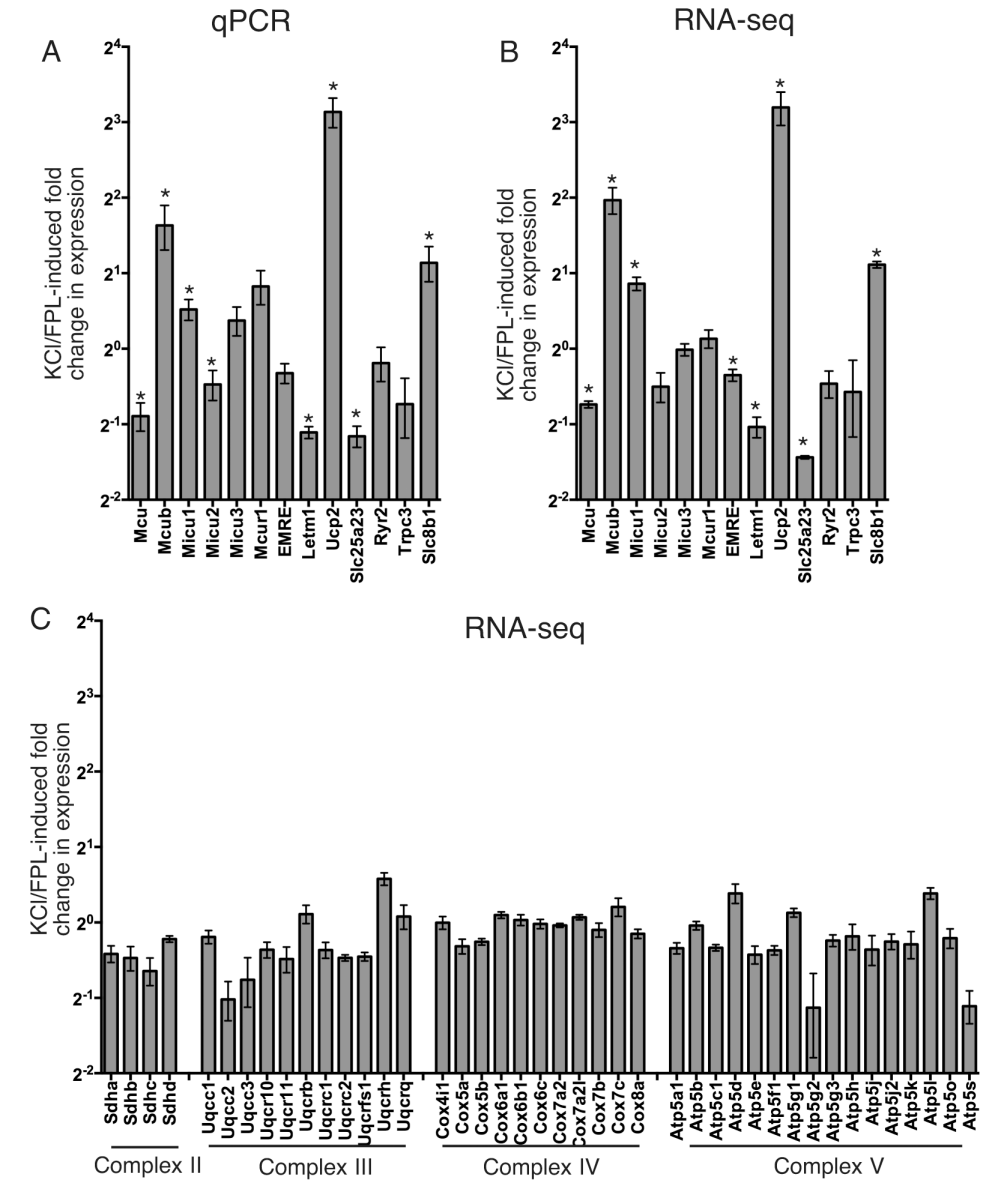
Ca^2+^ influx into cortical neurons modifies the expression of many MCRGs. **A)** Neurons were treated ± KCl/FPL64176/MK-801 (see methods) for 24h, after which MCRG mRNA expression was analysed by qRT-PCR, normalised to Gapdh, and the % change relative to untreated control cells calculated. *p values (left to right) are 0.0003, 0.029, 0.016, 0.047, 0.0002, 0.007, 0.001, 0.042; alpha = 0.05, Student's one-sample t-test (n = 4 except Mcu, Micu1 (n = 8), Mcub. Micu2 (n = 5) and Micu3 (n = 6)). **B)** Neurons were treated as in (A) and RNA-seq analysis of transcriptome changes performed, with MCRG fold changes shown here. * Exact p values (left to right) are 0.002, 0.025,0.017, 0.038, 0.008, 0.028, 6.4E-05, 0.003; alpha = 0.05; Student's one-sample t-test (n = 3). **C)** KCl/FPL-induced changes in expression of a selection of nuclear encoded mitochondrial proteins (components of ETC complexes II-V), analysed by RNA-seq (n = 3).

We previously showed that the expression of Mcu was subject to transcriptional repression by action potential bursting [8], and that cytoplasmic Ca^2+^ influx was less tightly coupled to mitochondrial Ca^2+^ uptake [8], however, other MCRGs were not analysed. We therefore wanted to investigate whether any of the MCRGs under study were subject to regulation. We studied responses to L-type Ca^2+^ channel activation, an important mediator of activity-dependent gene regulation [34–38]. Cortical neurons were subject to KCl-induced membrane depolarization in the presence of the L-type Ca^2+^ channel agonist FPL64176 (KCl/FPL), which leads to robust and uniform Ca^2+^ responses [39]. Consistent with our previous study, we found that KCl/FPL-induced Ca^2+^ influx triggered a 2-fold reduction in *Mcu* expression (Fig 3). Interestingly, expression of another gene implicated in mitochondrial Ca^2+^ uptake, *Letm1*, was also repressed, while a third, *Ucp2*, was induced (Fig 3). Other notable changes include repression of *Slc25a23*, and induction of *Mcub* (Fig 3).

To investigate Ca^2+^-induced MCRG mRNA expression using an alternative technique we performed RNA-seq on samples taken from neurons treated ± KCl/FPL as in Fig 3A. We observed a qualitatively similar relative gene expression pattern using RNA-seq as with the qPCR technique (Fig 3B). We also exploited the RNA-seq data set to look at the nuclear-encoded components of mitochondrial electron transport chain (ETC) complexes II-V (Fig 3C). As can be seen, overall there are much smaller changes in the expression levels of these genes at the mRNA level after KCl/FPL treatment, compared to changes in MCRG expression. Collectively, both qPCR and RNA-seq show that the changes in MCRG mRNA expression by Ca^2+^ influx are quite widespread, raising the possibility that Ca^2+^ signalling not only leads to changes in the coupling of cytoplasmic Ca^2+^ influx to mitochondrial Ca^2+^ uptake, but also lead to changes in the relative importance of the proteins involved.

### RNA-Seq Reveals Wide Range of MCRG mRNA Expression

**Fig 4:**
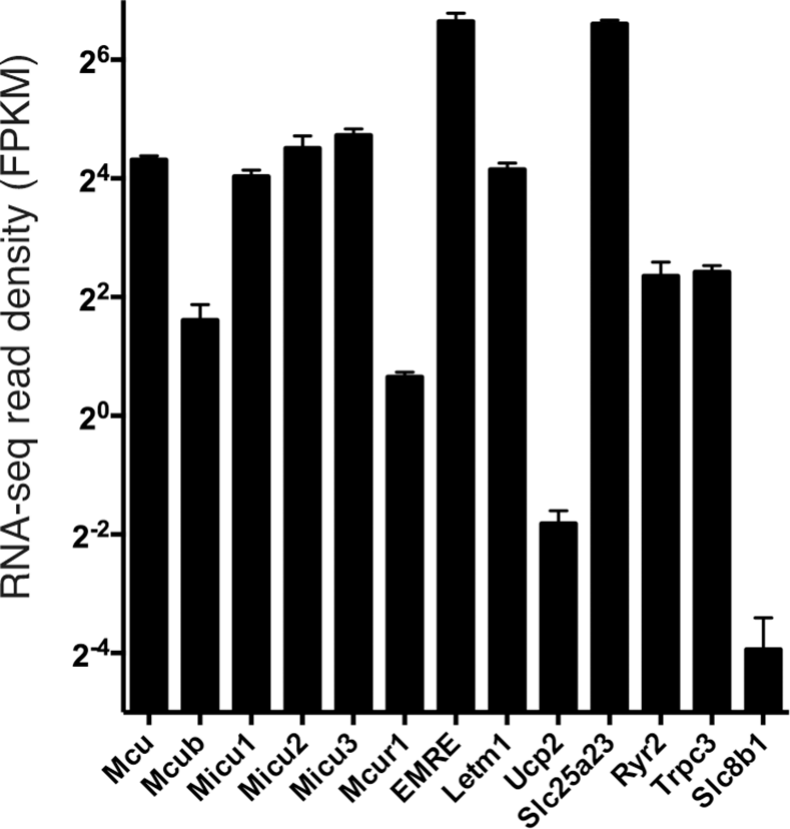
RNA-seq read density coverage of MCRGs in astrocyte-free cortical neurons. See methods for experimental details. n = 3 biological replicates.

Although the above data provide information about the relative expression of a specific gene's mRNA under different conditions or in different cell types, they do not provide any information regarding the relative expression of the different MCRGs compared to each other. For example, what is the relative expression of *Mcu* and *Mcub* mRNAs in cortical neurons? An advantage of RNA-seq data is that a measure of relative inter-gene expression can be obtained by calculating for each gene the FPKM coverage (Fragments Per Kilobase Of Exon Per Million Fragments Mapped). The results revealed a wide range of read coverage, indicating a wide range of mRNA expression levels (Fig 4). Interestingly, while expression of uptake genes *Mcu* and *Letm1* were fairly similar, that of *Ucp2* was lower (Fig 4). The mitochondrial Ca^2+^ extrusion gene *Nclx/Slc8b1* was also expressed weakly at the mRNA level. Also of note, the relatively weak expression of *Slc8b1*, *Ucp2* and *Mcur1* appears to be neuron-specific, since expression of these genes is higher in astrocytes (Fig 1).

Another feature apparent from the RNA-seq data is the relatively low expression of *Mcub* (*Ccdc109b*) compared to Mcu itself (Fig 4). Mcub can form a multimeric complex with Mcu and act in a dominant negative manner [12]. However, its apparent low expression at the mRNA level suggests that in developing cortical neurons it may not have a strong influence on mitochondrial Ca^2+^ uptake. As a preliminary investigation we studied mitochondrial Ca^2+^ uptake in neurons cultured from *Mcub/Ccdc109b* knockout embryos, in comparison to neurons cultured from their wild-type littermates, using the mitochondrial matrix-targeted indicator mtGCaMP2. The Mcub^+/-^ mice crossed to generate these embryos were created by the International Mouse Phenotyping Consortium (IMPC). We confirmed that the *Mcub*^–/–^neurons were deficient in *Mcub* mRNA (Figure a in S1 Fig) and also that there was no compensatory adjustment to expression of MCU itself (Figure b in S1 Fig). We found that *Mcub*-deficient neurons had similar basal and NMDA-evoked mitochondrial Ca^2+^ levels compared to wild-type (Figs c-f in S1 Fig). These data suggest that in developing cortical neurons, Mcub may not strongly determine mitochondrial Ca^2+^ uptake. However, further experiments are required to establish this definitively, such as measurements of cytoplasmic Ca^2+^ influx and Ca^2+^ handling, as well as using a variety of stimuli of different intensities.

## Discussion

Within this study we have taken a first step towards understanding the factors which can influence the expression of genes involved in mitochondrial matrix Ca^2+^ influx and eflux. We focussed on mRNA expression, which enabled us to screen a larger number of genes over multiple conditions. However, it is important to note that relative differences in mRNA are not always reflected in differences at the protein level. In the future it will be important to confirm the key differences reported above, at the protein level, although for some MCRGs there are currently no robustly validated antibodies available.

The relative density of mapped RNA-seq reads per kb of mRNA gives a useful indication of the level of mRNA expression. The relatively low expression of Nclx (Slc8b1) in cortical neurons is an interesting observation which requires further investigation to determine if these cells have below-average NCLX-dependent mitochondrial Ca^2+^ extrusion capacity. Another interesting observation was the low level of *Mcub* expression compared to *Mcu*. However, it should be noted that in the original paper describing the dominant negative influence of MCUB, tissue expression levels measured by qPCR (and normalized to a housekeeping gene) were also found to be lower than *Mcu* [12]. Nevertheless, certain tissues, such as the lung, appeared to have a *Mcub*:*Mcu* ratio higher than others, for example, skeletal muscle [12]. Thus, it could be that the effects of Mcub-deficiency are more apparent in cells from certain tissues than others.

Another advantage of gaining metrics on relative gene expression is that they can put dramatic fold-changes in gene expression into context. For example, at first glance the increase in *Ucp2* expression triggered by KCl/FPL-induced Ca^2+^ influx appears striking (Fig 3). However, the functional significance of this is unclear given the apparently low basal expression of *Ucp2* (Fig 4). In a similar vein, the tendency for *Mcub* expression to increase may have limited functional impact on the cell given its low basal expression. Likely to be of more functional relevance is the influence of Ca^2+^ signaling on *Letm1* expression, which is repressed in a very similar manner to *Mcu*, and so may contribute to the reduction in cytoplasm-mitochondria Ca^2+^ coupling seen in response to synaptic activity [8]. The modest, but significant increase in *Micu1* by KCl/FPL stimulation could also potentially influence MCU function. The weaker coupling of cytoplasmic Ca^2+^ influx to mitochondrial Ca^2+^ uptake [8] may reflect a homeostatic feedback control to limit mitochondrial overload. Synaptic activity is known to induce other types of homeostatic response, such as in synaptic strength or intrinsic properties, or neurotransmitter uptake and antioxidant capacity [40–42]. It would not be surprising if analogous mechanisms applied to mitochondrial Ca^2+^ handling.

Another outstanding issue regarding MCRG regulation is whether the type of Ca^2+^ signal determines the magnitude or even direction of change. Ca^2+^ signals induced by membrane depolarization or synaptic activity are generally neuroprotective [43–45], while chronic activation of NMDARs, including extrasynaptic ones, promotes neurodegeneration [46–48]. Synaptic and extrasynaptic NMDAR activation leads to distinct transcriptional responses that contribute to these differential effects [30, 49–51]. It will be of interest to investigate whether MCRGs are differentially regulated by synaptic vs. extrasynaptic NMDAR activity.

The difference in Mcu expression between CA1 and CA3 regions of the adult hippocampus was the most notable result from the MCRG analysis of these regions (Fig 2). The increased expression of Mcu in the CA3 region at the mRNA and protein level is in accordance with the in situ hybridisation data for Mcu on the Allen Mouse and Human Brain Atlases. This raises the possibility that the coupling of cytoplasmic Ca^2+^ influx to mitochondrial Ca^2+^ uptake is different in these two regions, although this will of course require direct investigation. The functional consequences of lower MCU expression for a neuron are likely to be pleiotropic. Efficient mitochondrial Ca^2+^ uptake may modify or buffer cytoplasmic Ca^2+^ transients, particularly in regions lacking other buffering organelles such as the ER. The presynaptic terminal is one such region where Ca^2+^ signals dictate the processes of vesicle endocytosis and exocytosis. Other consequences of efficient mitochondrial Ca^2+^ uptake in CA3 could be a tight coupling of activity-dependent Ca^2+^ influx to mitochondrial energy production via increased flux through the TCA cycle. On the other hand, CA3 mitochondria may be more vulnerable to Ca^2+^ overload. Given that CA1 and CA3 hippocampal regions have differential vulnerability to both oxidative stress and ischemia [52, 53] it is possible that altered Mcu expression may be a contributor or adaptive response to this differential vulnerability. Of course, cells that express less MCU may have a more significant MCU-independent component of Ca^2+^ uptake. Efficient viral knockdown of MCU reduces, but does not abolish, mitochondrial Ca^2+^ uptake following NMDAR activation in cultured hippocampal neurons [8], raising the likelihood of other mechanisms of Ca^2+^ uptake in neurons.

While expression of Mcu in neocortical neurons and astrocytes were found to be similar, the profile of Micu family expression was different. Astrocytes expressed more *Micu1* and *Micu2*, the two ubiquitously expressed Micu family members, while cortical neurons expressed more *Micu3*, a gene whose expression is restricted to neural tissue and skeletal muscle [16]. Since the role of MICU3 in MCU regulation is currently unclear, the effect of its expression on neuronal MCU-mediated Ca^2+^ uptake awaits clarification. Other key differences in MCRG expression between neurons and astrocytes centred on genes expressed at low levels in neurons, principally *Ucp2*, *Mcur1* and *Slc8b1*.

To conclude, the expression profile of MCRGs at the mRNA level shows considerable variability dependent on cell type, subtype and Ca^2+^ signalling. These factors may potentially influence the mechanism and nature of mitochondrial Ca^2+^ influx, as well as the downstream cellular consequences of this.

## Methods

### Cell Culture and Stimulations

All procedures were authorized under a UK Home Office approved project licence number 60/0556 and adhered to regulations specified in the Animals (Scientific Procedures) Act (1986). The work was approved by the University of Edinburgh Local Ethical Review Committee. Cortical cells were cultured from E17.5 CD1 mouse embryos, Ccdc109b^–/–^and wild type embryos (c57Blk/6), essentially as previously described [42]. Briefly, embryos were culled by Schedule 1 decapitation, cortices were dissociated in papain for 2 x 20 minutes and plated at a density of between 9–13 x 10^4^ neurons per cm^2^. Three cell culture types were prepared: Mixed [54] neuronal/astrocyte cultures (90% NeuN+ neurons and 10% GFAP+ astrocytes), highly enriched Neuronal [29] cultures (>98% NeuN+ neurons and <0.2% GFAP+ astrocytes) and highly enriched Astrocyte [55] cultures (>96% GFAP+ astrocytes). Mixed neuronal/astrocyte cultures and highly enriched neuronal cultures were prepared in Neurobasal growth medium plus 1% rat serum (Harlan Laboratories), B27 (Life Technologies Ltd), 1mM glutamine and 1x antibiotic/antimycotic (Life Technologies Ltd), while astrocyte cultures were obtained by plating cells at low density in Dulbecco's Modified Eagle Medium (DMEM) + 10% Fetal Bovine Serum and 1x antibiotic/antimycotic (all Life Technologies Ltd). To prevent astrocyte proliferation in neuron containing cultures, the anti-mitotic drug Cytosine β-D-arabino- furanoside hydrochloride (1.2mM) was applied either immediately post-plating (pure neuronal cultures) or on DIV4 (mixed cultures). Cultures were utilised as indicated between DIV3-17, and were fed with the above described appropriate growth medium on DIV4. Prior to experimentation cells were removed from growth medium and washed and placed in a minimal defined medium [56] containing 10% Minimum Essential Media (MEM, Life Technologies Ltd) and 90% Salt-Glucose-Glycine (SGG) medium, which is comprised of 114 mM NaCl, 0.219% NaHCO_3_, 5.292 mM KCl, 1 mM MgCl_2_, 2 mM CaCl_2_, 10 mM HEPES, 1 mM Glycine, 30 mM Glucose, 0.5 mM sodium pyruvate, 0.1% Phenol Red; osmolarity 325 mosm/l) for at least 3 h. KCl/FPL stimulations were performed as described previously [39, 57, 58]. KCl/FPL stimulation involved neurons being exposed to 50 mM KCl by adding 0.41 volumes of KCl depolarization solution (10 mM HEPES, pH 7.2, 170 mM KCl, 1 mM MgCl_2_, 2 mM CaCl_2_) in the presence of 5 μM FPL64176 (Tocris), plus an NMDA receptor antagonist (MK-801, 5 μM) to prevent any excitotoxicity.

### Mcub/Ccdc109b-Deficient Mice

Ccdc109b-tm1a mice were derived from C57BL6/NTac ES cells [59] and generated at MRC Harwell as part of the International Mouse Phenotyping Consortium. To create the null allele (tm1b), an IVF was carried out using ccdc109b^tm1a/+^ sperm and C57BL6/NTac oocytes. Two cell ccdc109b^tm1a/+^ embryos were converted to ccdc109b^tm1b/+^ by addition of soluble cell permeable cre (TAT-Cre (Tat-NLS-Cre, HTNC, HTNCre), Excellegen, Rockville, USA). This conversion occurs by cre excision of the selection cassette and exon 2 of the ccdc109b gene making a null allele. Following washing to remove the soluble cre the IVF procedure was completed as normal. The ccdc109b^tm1b/+^ were crossed to C57BL6/NTac and then intercrossed to create ccdc109b^tm1b/tm1b^, ccdc109b^tm1b/+^ and ccdc109b^+/+^ embryos. For further details on the allele see:

http://www.mousephenotype.org/data/alleles/MGI:1914065/tm1a%28KOMP%29Mbp. Genotyping primers used were as follows: Fwd: GGC CAT GGC AAC TTA CAA AA' Rev1: GCC TGT TGG AGG AGA GAT GT; Rev2: GAA CTT CGG AAT AGG AAC TTC G. WT band: 253nt; KO band: 147nt.

### RNA Isolation, RT-PCR and qPCR

RNA isolation from cultures was performed essentially as described [60]. Briefly, RNA was isolated using the Roche High Pure RNA Isolation Kit (including optional DNase treatment), according to manufacturer’s instructions (Roche, Hertfordshire, UK). For isolation of RNA from the adult hippocampus, the CA1 and CA3 regions of the hippocampus were dissected from brain slices of adult mice. RNA was isolated using the Roche High Pure RNA Tissue Kit (including optional DNase treatment), according to manufacturer’s instructions (Roche, Hertfordshire, UK). The hippocampal regions in the Roche lysis buffer were homogenized using the QIAGEN QIAshreddar (QIAGEN Ltd., Manchester, UK), before being placed into the Roche High Pure RNA Tissue Kit columns. For qPCR, cDNA was synthesized from 1–3 μg RNA using the Roche Transcriptor First Strand cDNA Synthesis Kit, according to manufacturer’s instructions, and as previously described [61]. cDNA was then stored at -20°C or immediately diluted (equivalent of 6 ng of initial RNA per 15 μl qPCR reaction, per gene of interest) for real-time PCR in a Stratagene Mx3000P QPCR System (Agilent Technologies, Waldbronn, Germany), using the Roche FS universal SYBR Green MasterRox mix, according to manufacturer’s instructions. The required amount of template was mixed with water, SYBR Green MasterRox mix and forward and reverse primers (200 nM each final concentration) to the required reaction volume. Technical replicates as well as no template and no RT negative controls were included and at least 3 biological replicates were studied per experiment. The qRT-PCR cycling programme was 10 min. at 95°C; 40 cycles of 30 sec. at 95°C, 40 sec. at 60°C with detection of fluorescence and 30 sec. at 72°C; 1 cycle (for dissociation curve) of 1 min. at 95°C and 30 sec. at 55°C with a ramp up to 30 sec. at 95°C (ramp rate: 0.2°C/sec) with continuous detection of fluorescence on the 55–95°C ramp. Data was analysed using the MxPro qPCR analysis software (Stratagene), with 18s or GAPDH expression utilized as an internal normaliser. Primer sequences are noted below in Table 1.

**Table 1:** 

Gene	Also known as	FWD	REV
*Mcu*	Ccdc109a	CGCCAGGAATATGTTTATCCA	CTTGTAATGGGTCTCTCAGTCTCTT
*Mcub*	Ccdc109b	CACTCCATTATGGGACAGTGG	CGACAACATCGGCTTGACTA
*Micu1*	Efha3, Cbara1	GAACTAGCTGTGGGCTCTCG	GGTGGCAAAATATCGGAAAA
*Micu2*	Efha1	ACATGACACCCCGAGACTTC	CGATCATCTCATTCCCATCC
*Micu3*	Efha2	AACTCCACCAGTTTGGAAGG	CACCATCTCATTTCCGTCAG
*Mcur1*	Ccdc90a	AGCCCTCAGAGCAGAAAATG	CCAGCATCTTCTTCTCGTTCA
*EMRE*	Smdt1	CCTGGGTTGCAGTTCGAC	GTACCTGGCTGAGACCTGAG
*Letm1*		TCCTGCGTTTCCAGCTCACCAT	GTCTTCTGTGACACCGAGAGCT
*Ucp2*	Slc25a8	TAAAGGTCCGCTTCCAGGCTCA	ACGGGCAACATTGGGAGAAGTC
*Slc25a23*		TTGATTGGCAGGAATGGCGAGAC	GTCAGGCATTCACCGATGTCCA
*Ryr2*		ACCTACTCCGAAGGCTGGTGTT	TTCTTCCGAGGCAGCACCAAAG
*Slc8b1*	Nclx, Slc24a6	GAGACCACTGTCCAGATCCTGA	GAGCAGCAACAAGAACTCCACAG
Uqcrc2		TGCTCCTCTGTCAAGAATCG	CAGTGTACGCCATGTTTTCC
Ndufs2		GGATCCGCTTGCCAAGTC	CTCAATGAGCTTCTCCGTGC
*Trpc3*	Trp3	GCATACTTGTCGCTGTCCAG	CGACTCAGTGACGCTTTGTG
*18s*		GTGGAGCGATTTGTCTGGTT	CAAGCTTATGACCCGCACTT
*Gapdh*		GGGTGTGAACCACGAGAAAT	CCTTCCACAATGCCAAAGTT

### Western Blotting

This was performed essentially as described [62]. Briefly, for protein extraction, cells were immediately lysed in NuPage LDS sample buffer and boiled at 100°C for 10 min. Approximately 30 μg of protein was loaded per lane and the gel then subjected to gel electrophoresis and western blotting using the Xcell Surelock system with precast gradient gels (4–20%, Life Technologies Ltd), according to manufacturer's instructions. Gels were blotted onto PVDF membranes, and blocked for 1 hour at room temperature with 5% (w/v) non-fat dried milk in TBS with 0.1% Tween 20. Membranes were incubated overnight at 4°C with primary antibodies diluted in the blocking solution: Anti-MCU (1:5000, Sigma), anti-UQCRC2 (Abcam 13G12, 1:30,000) anti-β-actin (1:200000, Sigma). For visualisation of Western blots, HRP-based secondary antibodies were used followed by chemiluminescent detection on Kodak X-Omat film. Appropriate exposures were taken such that bands were not saturated. Where required a stripping agent was utilized in order to enable detection of the same blot with a second antibody, as achieved through the use of Re-Blot Plus Stripping Solution (Millipore). Western blots were analyzed by digitally scanning the blots, followed by densitometric analysis (ImageJ). Loading was normalised via amounts of Mcu relative to UQCRC2 or β-actin as loading control (on the same blot). For figure preparation of example western blots, linear adjustment of brightness/contrast was applied (Photoshop) equally across the entire image, taking care to maintain some background intensity.

### RNA Sequencing

Total RNA from mouse cortical neurons was assessed for quality (Agilent Bionalyzer) and quantity (Invitrogen Qubit) before library preparation. Illumina libraries were prepared from 1 μg of total RNA using TruSeq RNA Sample Prep Kit v2 with a 10 cycle enrichment step as per the manufacturer's recommendations. Final libraries were pooled in equimolar proportions before Illumina sequencing on a HiSeq 2500 platform using 50 base paired-end reads. Raw reads were processed using RTA 1.17.21.3 and Casava 1.8.2 (Illumina).

Reads were mapped to the primary assembly of the mouse reference genome contained in Ensembl release 80. Alignment was performed with STAR, version 2.4.0i [63], with the option “—outSAMstrandField” set to “intronMotif” and the option “—outFilterIntronMotifs” set to “RemoveNoncanonical”, as recommend for the compatibility of STAR with Cufflinks; otherwise default values were used for all options. Gene expression was then estimated with Cufflinks, version 2.2.1 [64, 65] using gene annotations from Ensembl release 80. Cufflinks was run in expression estimation mode only (-G flag), and corrections for multi-read mapping (-u flag) and bias (-b flag) were enabled; otherwise default values were used for all options. Estimates of gene mRNA expression were then extracted in units of fragments per kilobase of exon per million mapped fragments.

### Mitochondrial Ca^2+^ imaging

This was performed essentially as described [66]. Neurons were transfected (Lipofectamine 2000) as previously described [67] with a mito-GCaMP2-encoding vector [68], the fluorescence signal of which was detected using a standard GFP filter set (ex 480±20; em 527±15). Changes in Ca^2+^ were expressed as (F-F_min_)/(F_max_-F) according to the equation [Ca^2+^] = Kd*(F-F_min_)/(F_max_-F). F_max_ was obtained when cells were treated with the cell-permeable Ca^2+^ ionophore ionomycin which both inserts into the plasma membrane and passes into the cell, inserting into mitochondrial membranes, leading to saturation of the indicator when in regular medium (2 mM Ca^2+^). F_min_ was obtained under the same conditions except in zero Ca^2+^ medium. The linear relationship between [Ca^2+^] and (F-F_min_)/(F_max_-F) has been previously confirmed by calibrating the indicator as expressed in neurons, exposing them to ionomycin in the presence of sequentially different solutions of precise [Ca^2+^], obtained by mixing K_2_EGTA and CaEGTA solutions (Calcium Calibration Buffer Kit, Invitrogen) at different ratios [8].

### Statistical Analysis

Paired t-tests were used to compare non-independent data pairs, while student t-tests were utilized when the two groups were not related. Error bars represent standard error of the mean.

## Supporting Information

S1 Dataset Data relating to the main manuscript. (XLSX)

S2 Dataset Data relating to S1 Fig. (XLSX)

S1 Fig File containing S1 Fig. (PDF)
