# Supplementary material for: Expression of mRNA Encoding Mcu and Other Mitochondrial Calcium Regulatory Genes Depends on Cell Type, Neuronal Subtype, and Ca2+ Signaling
Source: PLoS One. 2016 Feb 1;11(2):e0148164. doi: 10.1371/journal.pone.0148164 (PMC4734683; doi:10.1371/journal.pone.0148164)
Supplement: S1 Fig — (PDF) [file pone.0148164.s003.pdf]

Fig. S1

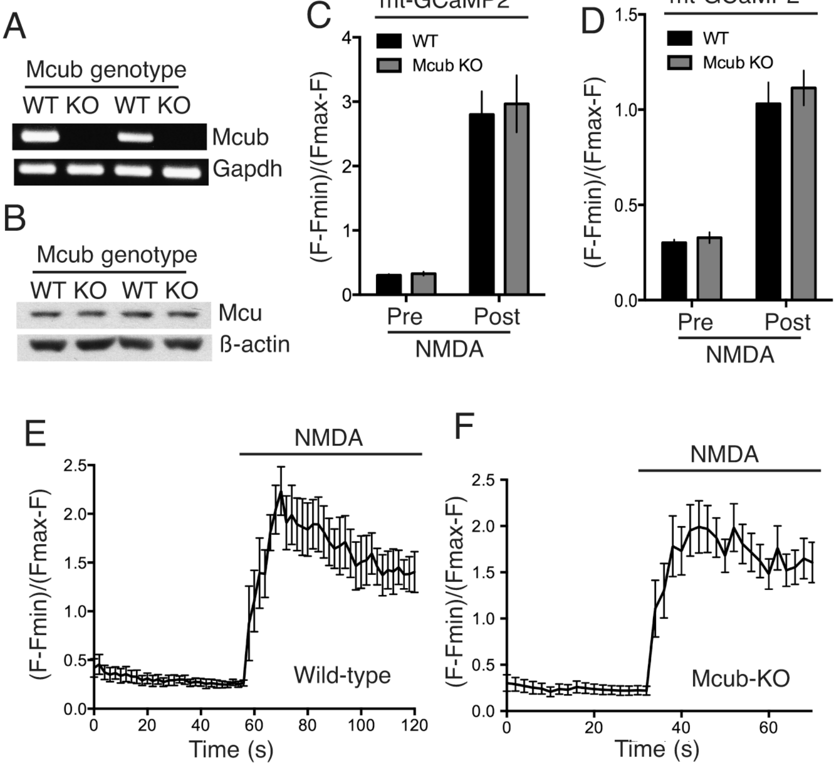

Fig. S1. A) qRT-PCR confirmation that Mcub-KO neurons do not express Mcub. **B)** Western blot to confirm that Mcub neurons do not have altered levels of Mcu. **C,D)** WT and Mcub-KO neurons (cultured from littermates) were transfected with mt-GCaMP2 and images taken before and after treatment with NMDA (20 µM). Mitochondrial Ca^{2+} levels were calculated as a multiple of the Kd: $(F-F_{min})/(F_{max}-F)$. Maximum (C) and average (D) Ca^{2+} levels were calculated before and after NMDA application; n=10 imaging experiments were performed for each genotype, within which 71 WT cells and 77 KO cells were measured. **E, F)** Example traces from individual experiments whose data contributed to 5C and 5D. In the WT example (E), the mean ± s.e.m. of 10 cells within that single experiment is shown. In the KO example (F), the mean ± s.e.m. of 6 cells within that single experiment is shown.
